# Developing a predictive model for high‐risk older adults and Age‐Friendly ED screening

**DOI:** 10.1002/alz70858_098554

**Published:** 2025-12-25

**Authors:** Karen A. Hauser

**Affiliations:** ^1^ The Permanente Medical Group, San Francisco, CA, USA

## Abstract

Learn how the Emergency Department and Kaiser Permanente Division of Research (DOR) created and implemented a high performing, novel Geriatric Emergency Department Screening Score (GED‐SS). This automated screening tool integrates clinical data from one of the US's most comprehensive, integrated community healthcare systems to facilitate real‐time, early identification of high risk older adult and dementia patients seen in the ED who would benefit from geriatric and cognitive assessments and care coordination. Discussion will include an overview of how the GED‐SS was developed, validated, and integrated into clinical care to predict utilization and mortality outcomes. Hear how the DOR and clinical operations team have now implemented this score into routine emergency care to better identify and target older patients at risk for geriatric syndromes and probable dementia, using this risk screening score to provide targeted transdisciplinary care for at risk patients while in the ED.

